# A novel avian isolate of hepatitis E virus from Pakistan

**DOI:** 10.1186/s12985-019-1247-0

**Published:** 2019-11-21

**Authors:** Tahir Iqbal, Umer Rashid, Muhammad Idrees, Amber Afroz, Saleem Kamili, Michael A. Purdy

**Affiliations:** 1 Department of Biochemistry and Biotechnology, University of Gujrat, Gujrat, 50700 Pakistan; 2 Division of Viral Hepatitis, Centers for Disease Control and Prevention (CDC), MS-A33, 1600 Clifton Rd NE, Atlanta, GA 30329 USA; 3 Centre for Applied Molecular Biology (CAMB), University of the Punjab, Lahore, 53700 Pakistan; 4 Hazara University, Mansehra, 21300 Pakistan

**Keywords:** Avian HEV, Novel strain, Layer chickens, Pattoki Punjab, Pakistan

## Abstract

**Background:**

Avian hepatitis E virus (aHEV) has been associated with hepatitis-splenomegaly syndrome (HSS) in chickens along with asymptomatic subclinical infection in many cases. So far, four genotypes have been described, which cause infection in chickens, specifically in broiler breeders and layer chickens. In the present study, we isolated and identified two novel aHEV strains from the bile of layer chickens in Pakistan evincing clinical symptoms related to HSS.

**Methodology:**

Histology of liver and spleen tissues was carried out to observe histopathological changes in these tissues. Bile fluid and fecal suspensions were used for viral RNA isolation through MegNA pure and Trizol method which was further used for viral genome detection and characterization by cDNA synthesis and amplification of partial open reading frame (ORF) 1, ORF2 and complete ORF3. The bioinformatics tools; Molecular Evolutionary Genetics Analysis version 6.0 (MEGA 6), Mfold and ProtScale were used for phylogenic analysis, RNA secondary structure prediction and protein hydropathy analysis, respectively.

**Results:**

Sequencing and phylogenetic analysis on the basis of partial methyltranferase (MeT), helicase (Hel) domain, ORF2 and complete ORF3 sequence suggests these Pakistani aHEV (Pak aHEV) isolates may belong to a Pakistani specific clade. The overall sequence similarity between the Pak aHEV sequences was 98–100%. The ORF1/ORF3 intergenic region contains a conserved *cis*-reactive element (CRE) and stem-loop structure (SLS). Analysis of the amino acid sequence of ORF3 indicated two hydrophobic domains (HD) and single conserved proline-rich domain (PRD) PREPSAPP (PXXPXXPP) with a single PSAP motif found in C-terminal. Amino acid changes S15 T, A31T, Q35H and G46D unique to the Pak aHEV sequences were found in the N-terminal region of ORF3.

**Conclusions:**

Our data suggests that Pak aHEV isolates may represent a novel Pakistani clade and high sequence homology to each other support the supposition they may belong to a monophyletic clade circulating in the region around Pakistan. The data presented in this study provide further information for aHEV genetic diversity, genotype mapping, global distribution and epidemiology.

## Background

The family *Hepeviridae*, contains genetically diverse hepatitis E virus (HEV) strains, and is currently divided into two genera – *Orthohepevirus* and *Piscihepevirus*. The genus *Orthohepevirus* is subdivided into four species; *Orthohepevirus A* (containing HEV from humans, wild boar, pig, mongoose, deer, rabbits and camels), *Orthohepevirus B* (avian HEV), *Orthohepevirus C* (rat, ferret, bandicoot, shrew and mink HEV) and *Orthohepevirus D* (bat HEV). The genus *Piscihepevirus* includes a single species with a single member from cutthroat trout. HEV strains detected in little egret, kestrel, moose and foxes are currently unclassified [1–3].

The aHEV was first isolated and identified from chickens suffering from HSS in the United States in 2001 [4]. Another chicken disease, Big Liver and Spleen (BLS), was also found to be associated with aHEV [5]. The genome of aHEV consists of a single-stranded positive sense RNA that is approximately 6.6 kb in length that is 600 bp smaller than *Orthohepevirus A* genomes. Like other HEV species, the aHEV genome consists of three open reading frames (ORFs). Non-structural genes necessary for viral replication (methyltransferase, putative papain-like cysteine protease, hypervariable region (HVR), macro domain, helicase and replicase), are encoded by ORF1. ORF2 encodes the capsid, and ORF3 codes for a small regulatory protein required for virion egress [6, 7]. The shortened portion of the aHEV genome is ORF1, which has a truncated region between MeT and the HVR [8], as compared to the *Orthohepevirus A* genome. So far, four aHEV genotypes (Gt) have been isolated from avians; Gt1 (Australia and Korea), Gt2 (USA), Gt3 (Hungary and China), and Gt4 (Taiwan and Hungary) [9]. Interestingly, all four genotypes belong to a single serotype, which may facilitate development of a vaccine effective against all four genotypes [10]. Apart from chickens, novel avian-like HEVs have been isolated from little egrets (*Egretta garzetta*) and a sparrow [8, 11]. No zoonotic risk has been associated with aHEV. Attempts to experimentally infect mice and rhesus macaques with aHEV have been unsuccessful [6, 12]. However, experimental infection in turkeys (*Meleagris gallopavo*) with aHEV was successful with subsequent passage to naive turkeys [13]. The isolation of aHEV genotypes 1 and 3 from 62 different wild bird species indicates a broad host range for aHEV in avians [14].

To the best of our knowledge, this is the first description of an aHEV from South Asia. The current study was designed to isolate and characterize aHEV from layer chickens in Pakistan, and we report a novel aHEV strain that appears to belong to a previously unknown Pakistani clade.

## Materials and methods

### Sampling and histology

Spleen and liver tissue, bile fluid, and fecal swabs were collected from 19 layer chickens (nos. PT1 – PT19) within 12 h after their death; age range 30–90 weeks, from different poultry farms situated in Pattoki Punjab, Pakistan (31°1 N 73°51E). These samples were collected with the help of a veterinarian after necropsy, and the carcasses were examined for clinical symptoms related to HSS; enlarged liver and spleen, serosanguinous fluid in the abdominal cavity and hemorrhagic foci in liver tissue. Liver and spleen tissues were fixed in 10% formalin for histological studies, embedded in paraffin and stained with hematoxylin and eosin (H&E) [15]. Bile fluid and fecal swab samples were collected in sterilized 1.5 ml microfuge tubes, transported to the lab on ice and stored at − 80 °C for future analysis. Fecal suspension samples positive for aHEV RNA were kindly provided by Dr. X. J. Meng (Center for Molecular Medicine and Infectious Diseases, Virginia Tech, VA, USA).

### Viral RNA isolation, detection, and gene amplification

Total RNA was isolated from bile and fecal suspensions using Trizol (Invitrogen) and the MegNA Pure 24 system (Roche), following manufacturers’ instructions. A total volume of 200 μl of either bile fluid or fecal suspension was used for total RNA extraction.

Primers for this study (Table 1) were designed from the conserved regions in the aHEV genome- identified by multiple sequence alignment (MEGA 6) [16] of complete aHEV genomes (AM943647, AY535004, AM943646, KF511797) available from GenBank. The detection of aHEV RNA in bile fluid and fecal suspension samples was carried out according to the procedure reported by Kwon et al. [17]. The partial helicase (Hel) and capsid gene sequences were amplified using primer sets RPHF/RPHR and RPO2F/RPO2R, respectively. The following thermal profile was used to amplify these fragments; initial denaturation at 95 °C for 2 min followed by 40 cycles denaturation at 95 °C for 30 s, annealing at 50 °C for 30 s and extension at 72 °C for 1 min. Fragments from the ORF1, ORF2 and ORF3 junction region, a partial MeT and an ORF2 fragment were amplified with primer sets APO31S/APO31A, aHCGF/F1R and F9F/aHCGR, respectively using the following conditions; initial denaturation at 95 °C followed by 40 cycles of denaturation at 95 °C for 45 s, annealing at 55 °C for 45 s and extension at 72 °C for 3 min. The PCR products were observed on 0.8, 1, and 2% agarose gels according to their size.

**Table 1:** Primers used in this study

Fragment	Primer ID	Sequence (5′-3′)	GC%	Nucleotide Position^a^	Fragment size (bp)	Reference
MeT	aHCGF	GCATGACCCCATGCCAGGGTAAG	61	1–23	849	This study
	F1R	CGGCATGGGGCAGGGCTGGGT	76	849–829		
Hel	RPHF	TGGCGCACYGTWTCYCACCG	60	2791–2810	186	[16]
	RPHR	CCTCRTGGACCGTWATCGACCC	59	2956–2977		
ORF2	RPO2F	GGTATGGTTGATTTTGCCATAAAG	38	5433–5456	280	[16]
	RPO2R	GCTGCNCGNARCAGTGTCGA	55	5694–5713		
ORF2	F9F	AATGGTAGCTCCGTGGTTTGGTATGC	50	6273–6298	385	This study
	aHCGR	ACTATGCCCGAGATGGGAGG	60	6658–6639		
ORF123 junction & complete ORF3	APO31S	ACCATCCAGCTTGTGGCGG	63	4480–4499	902	This study
	APO31A	CACAAACCATGAGCATGCCGGACG	58	5382–5359		

^a^nucleotide positions using reference sequence AM943646

### Sequencing and phylogenetic analysis

Bile and fecal specimens testing positive for HEV RNA by RT-PCR were sequenced bi-directionally by Sanger sequencing using the BigDye Terminator v3.1 Cycling Sequencing kit (Applied Biosystems). The sequences were analyzed using DNASTAR (Lasergene). A BLAST search was done on the obtained sequences [18]. Percent identity (PI) and phylogenetic analyses was done with MEGA 6 and sequence alignments were done using ClustalX2.1 in comparison with other aHEV (*Orthohepevirus B*) (Table 2) and *Orthohepevirus A* sequences (Table 3) [16, 19]. The RNA Stem-loop structure (SLS) prediction was done on the Mfold web server using a folding temperature fixed at 37 °C [20].

**Table 2:** Percent similarity of Pak aHEV strains with other *Orthohepevirus B* (avian) sequences based on partial ORF1 (MeT, Hel), ORF2 and complete ORF3 nucleotide sequences

*Orthohepevirus B* sequences Genotype/ country (GenBank accession no.)	% nucleotide similarity			
	MeT	Hel	ORF2	ORF3
Pak aHEV (PT12B)				
Gt1/Australia (AM943647)	**90**^a^	83	84	**95**
Gt1/Korea (JN597006)	88	84	83	94
Gt2/USA (AY535004)	88	84	**85**	94
Gt3/ Hungary (AM943646)	87	**85**	84	94
Gt3/China (GU954430)	87	84	84	94
Gt4/ Taiwan (KF511797)	87	80	83	93
Pak aHEV (PT16B)	98	100	99	100
Pak aHEV (PT16B)				
Gt1/Australia (AM943647)	**91**	83	**84**	**95**
Gt1/Korea (JN597006)	89	84	83	94
Gt2/USA (AY535004)	89	84	84	94
Gt3/ Hungary (AM943646)	88	**85**	83	94
Gt3/China (GU954430)	88	84	84	94
Gt4/ Taiwan (KF511797)	88	80	83	93
Pak aHEV (PT12B)	98	100	99	100

^a^Bolded numbers show the highest sequence identity to the Pak aHEV sequences. Korea, Republic of Korea

**Table 3:** Percent similarities of Pak aHEV sequences with *Orthohepevirus A* sequences based on partial ORF1 (MeT, Hel), ORF2 and complete ORF3 nucleotide sequences

mHEV strains Genotype/ country (GenBank accession no.)	% nucleotide similarity			
	MeT	Hel	ORF2	ORF3
Pak aHEV (PT12B)				
Gt1/China (D11092)	57	**66**^a^	46	42
Gt1/Pakistan (M80581)	55	66	**49**	42
Gt1/Burma (M73218)	58	64	48	42
Gt3/USA (AF082843)	56	61	48	41
Gt3/China (FJ527832)	55	61	46	42
Gt4/China (AJ272108)	55	60	47	**45**
Gt4/Korea (FJ763142)	58	61	47	43
Gt2/Mexico (M74506)	**59**	65	47	42
Pak aHEV (PT16B)				
Gt1/China (D11092)	56	**66**	47	42
Gt1/Pakistan (M80581)	55	66	**50**	42
Gt1/Burma (M73218)	57	64	48	42
Gt3/USA (AF082843)	55	61	48	41
Gt3/China (FJ527832)	55	61	46	42
Gt4/China (AJ272108)	55	60	47	**45**
Gt4/Korea (FJ763142)	**58**	61	47	43
Gt2/Mexico (M74506)	58	65	47	42

^a^Bolded numbers show the highest sequence identity to the Pak aHEV sequences. Korea, Republic of Korea

### Protein hydropathy analysis

The ProtScale tool [21] on the ExPASy server (https://web.expasy.org/protscale/) was used to analyze the hydropathic character of the ORF3 peptide using the Kyte and Doolittle scale [22].

## Results

Out of a total of 19 layer chickens, aHEV RNA was detected in 2 bile samples (PT12B and PT16B). None of the fecal samples were positive for aHEV RNA. The aHEV RNA-positive bile samples came from 71 week old layer chickens with clinical symptoms related to HSS from the same poultry farm. Sequence information was obtained from these two aHEV RNA positive bile samples from five sub-genomic regions. Regions 40–447 nt (reference AM943646) and 2771–2956 nt cover part of the MeT and Hel domains in ORF1, respectively. The remaining three regions were concatenated together, and cover junction region between ORF1, ORF2 and the complete ORF3 sequence (4545–5475 nt) plus two additional fragments from ORF2 (5479–5829 nt and 6273–6658 nt). These aHEV sequences were submitted to GenBank (accession numbers: MG692742, MG692743, MG692744, MH018052, MH018053, MH094852, MH094853, MH243320 and MH243321).

### Pathologic examination

**Fig. 1:**
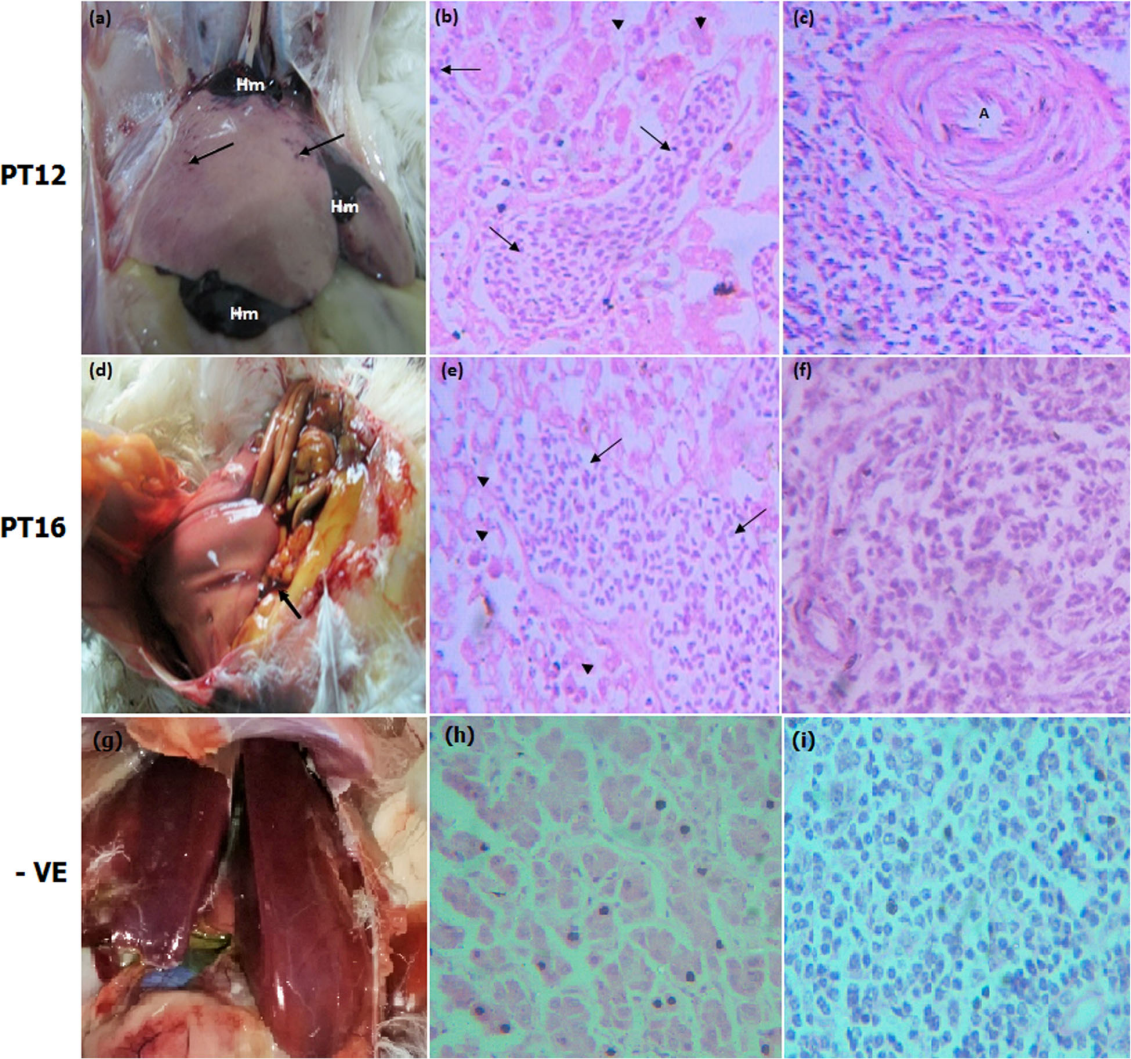
Histopathology of liver and spleen of Pak aHEV RNA positive layer chickens (PT12 upper panel, PT16 middle panel) and Pak aHEV RNA negative chicken (−VE = negative control, lower panel). **a** Enlarged hemorrhagic liver (Hm) with discoloration and multiple hemorrhagic foci on the surface (arrows), **d** Enlarged liver with discoloration and serosanguinous fluid in the abdominal cavity (arrow), (**g**) liver of Pak aHEV negative chicken. (**b** & **e**) Necrotizing hepatitis (arrow heads) with infiltration of lymphocytes (arrows), (**h**) liver negative control. (**c** & **f**) Depletion and aggregation of lymphocytes in the periarterioral sheaths (PAS), A = artery, (**i**) spleen negative control. H&E 40X

The gross pathological examination revealed enlargement and discoloration of the livers in both birds with hemorrhage and hemorrhagic foci in PT12 (Fig. 1a) and serosanguinous fluid in the abdominal cavity in PT16 (Fig. 1d). Liver histology showed severing necrotizing hepatitis with prominent infiltration of lymphocytes (Fig. 1b & e). There was also a depletion of lymphocytes in the spleens along with aggregates of lymphocytes, particularly in the periarteriolar sheath (PAS) areas (Fig. 1c & f).

### Sequencing and phylogenetic analysis

Sequence analysis indicated that both aHEV sequences shared the following nucleotide sequence identities (NSI) in the partial MeT (87–91%), Hel (83–85%), ORF2 (83–85%) and complete ORF3 (93–95%) when compared with homologous aHEV (Gt1–4) genome sequences, and both Pakistani sequences shared 98–100% sequence identity with each other. The Pakistani aHEV sequences showed high NSI when compared to the known aHEV genotypes with PT12B exhibiting the highest similarities to MeT (90%, Gt1), Hel (85%, Gt3), ORF2 (85%, Gt2) and ORF3 (95%, Gt1), and for PT16B the highest similarities were MeT (91%, Gt1), Hel (85%, Gt3), ORF2 (84%, Gt1 and 3) and ORF3 (95%, Gt1) (Table 2). Interestingly, amino acid sequence analysis using the complete ORF3 sequence showed that both Pak aHEV shared sequence identities of 89–92% with Gt1–4 avian HEV sequences. Both sequences shared overall NSIs for the partial MeT (55–59%), Hel (60–66%), ORF2 (46–60%) and complete ORF3 (41–45%) with homologous genome regions from *Orthohepevirus A* Gt1–4 (Table 3). The amino acid sequence identity between the Pak aHEV complete ORF3 sequences and corresponding *Orthohepevirus A* Gt1–4 sequences was 20–22%.

**Fig. 2:**
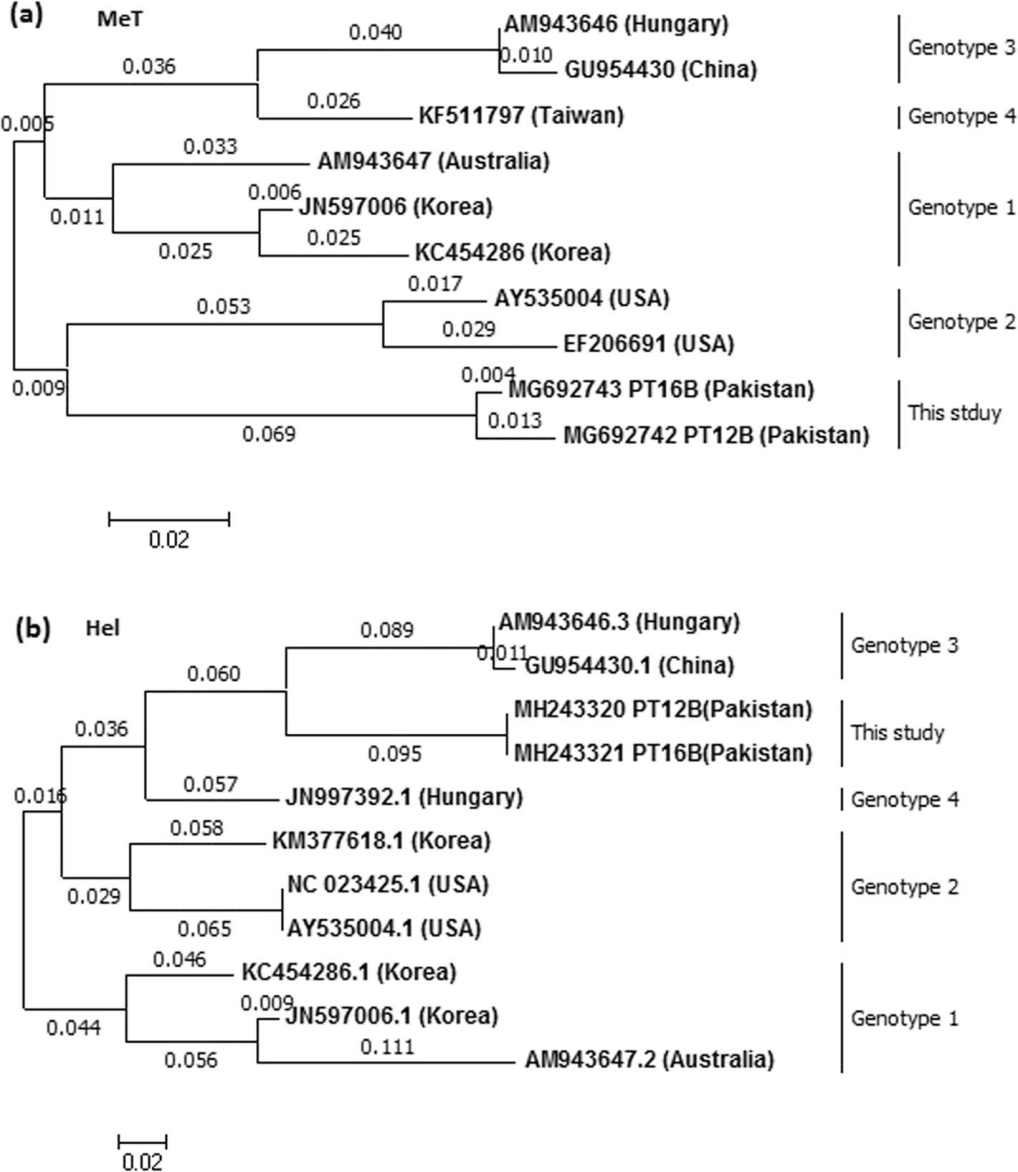
Phylogenetic relationship of ORF1 sequences from Pak aHEV isolates (this study) with other aHEV isolates established by maximum likelihood method, numbers on the branches represent branch lengths. **a** Based on partial MeT nucleotide sequence. **b** Based on partial Hel nucleotide sequence. GenBank accession number (country) is shown for each member. Pakistani sequences also contain the ID for the chicken from which the sequence was obtained. Korea, Republic of Korea

**Fig. 3:**
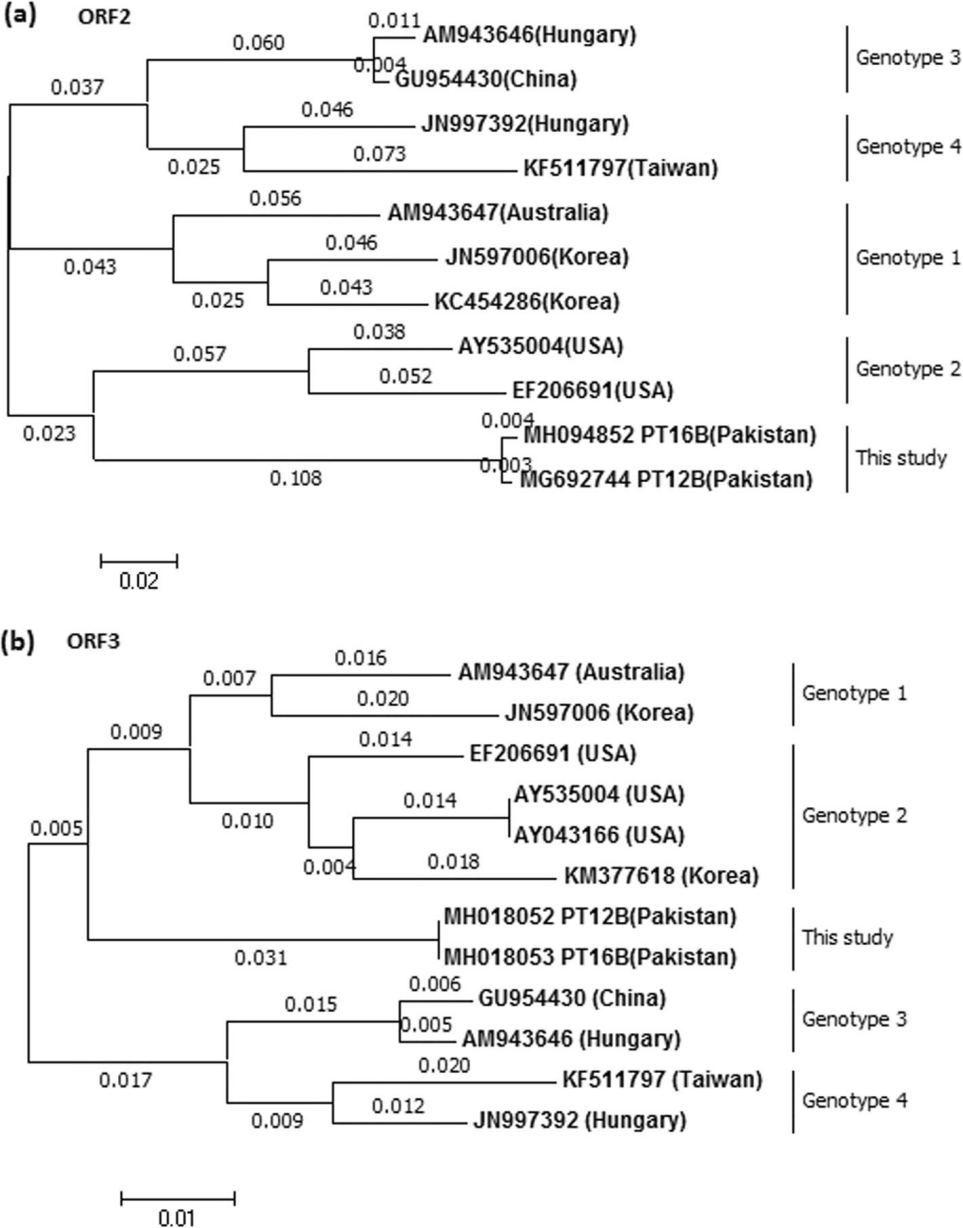
Phylogenetic relationship of ORF2 and ORF3 sequences from Pak aHEV isolates (this study) with other aHEV isolates established by maximum likelihood method, numbers on the branches represent branch lengths. **a** Based on partial ORF2 nucleotide sequence. **b** Based on complete ORF3 nucleotide sequence. GenBank accession number (country) is shown for each member. Pakistani sequences also contain the ID for the chicken from which the sequence was obtained. Korea, Republic of Korea

**Fig. 4:**
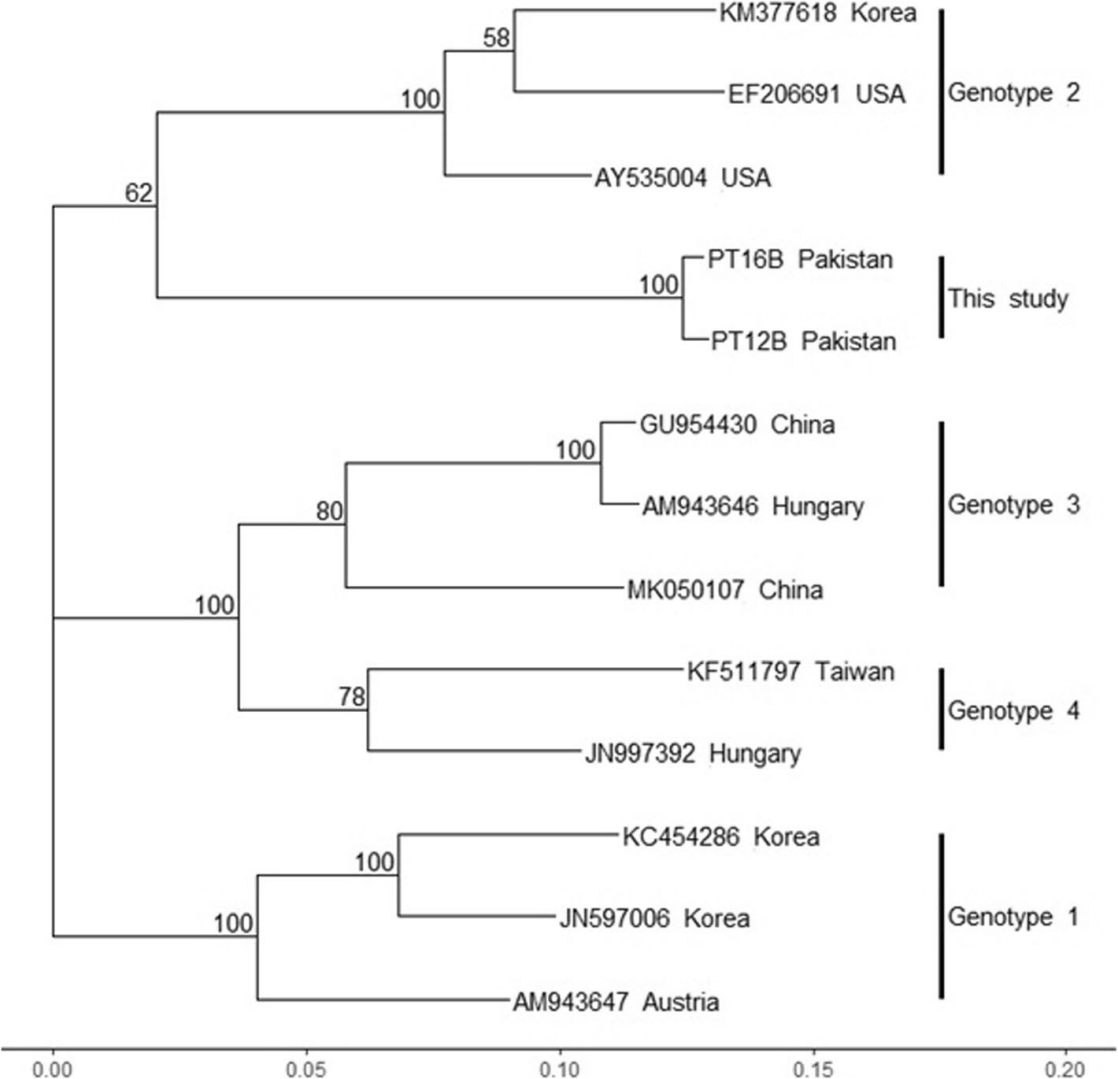
Maximum likelihood tree using concatenated MeT, Hel, ORF2 and ORF3 sequences from Pakistani aHEV sequences (this study) and other aHEV sequences. The scale bar at the bottom of the tree shows branch length. Node numbers are bootstrap values from 1000 replicate runs. GenBank accession number and country is given for each sequence. Because the Pakistani sequences are concatenated they are shown as the identifier for the animal from which the sequences were obtained. Korea, Republic of Korea

Analysis of phylogenetic trees using avian HEV sequences from the partial MeT, Hel, concatenated ORF2 and complete ORF3 nucleotide sequences, indicated that the Pakistani sequences cluster together, but do not cluster with any of the aHEV GT1–4 sequences, and the aHEV Gt1–4 sequences clustered into genotypic specific clades (Figs. 2 and 3). Figure 4 shows a bootstrap analysis for the fully concatenated Pakistani sequences. The figure shows that the Pakistani sequences form a monophyletic clade with high bootstrap support.

**Fig. 5:**
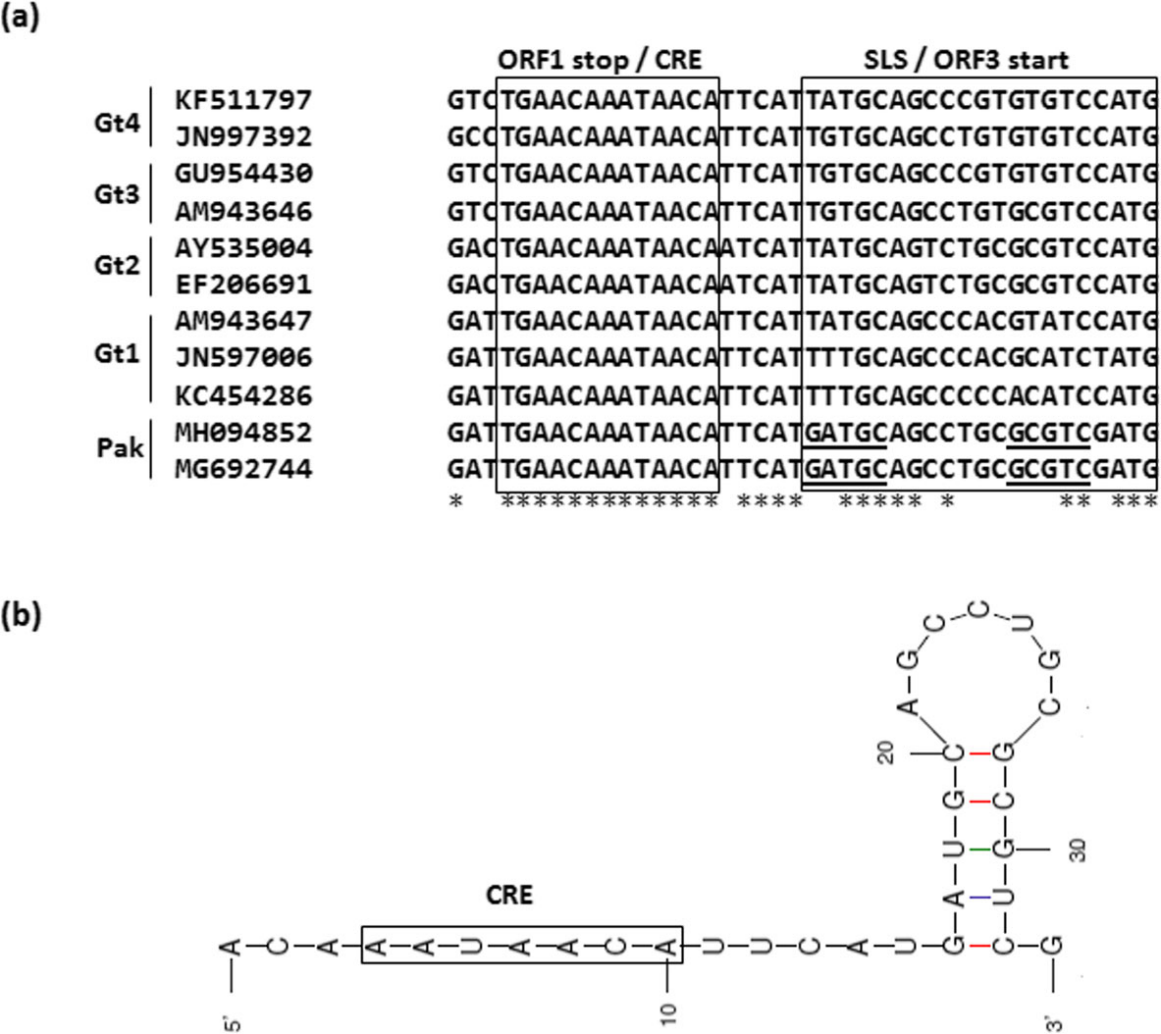
ORF1/ORF3 intergenic junction region. **a** Multiple sequence alignment showing the highly conserved *cis*-reactive element (CRE) and stem-loop structure (SLS). Predicted sequences (underlined) of Pak aHEV strains (MH094852, MG692744) involved in stem formation of SLS. * denotes conserved nucleotides positions. Genotypes are given for all used HEV strains. **b** Structure of SLS predicted in Pak aHEV sequences with Mfold program (∆G = − 5.90 kcal/mol)

Multiple sequence alignment of the ORF1/ORF3 intergenic junction region (IJR), 4600–4633 nt (reference AM943646), indicated sequence conservation *UGA*ACA**A**_4603_**AUAACA** of the ORF1 stop codon (Italic) and the *cis*-reactive element (CRE) (in bold). The second conserved region **G**_4616_**A*UGC****AG*C*C*UGC**GCG*UC***G_4633_, is involved in formation of a putative stem-loop structure. The bold bases indicate the bases involved in stem formation in Pak aHEV sequences, while Italic bases are conserved in all aHEV sequences (Fig. 5).

### Complete ORF3 amino acid sequence analysis

**Fig. 6:**
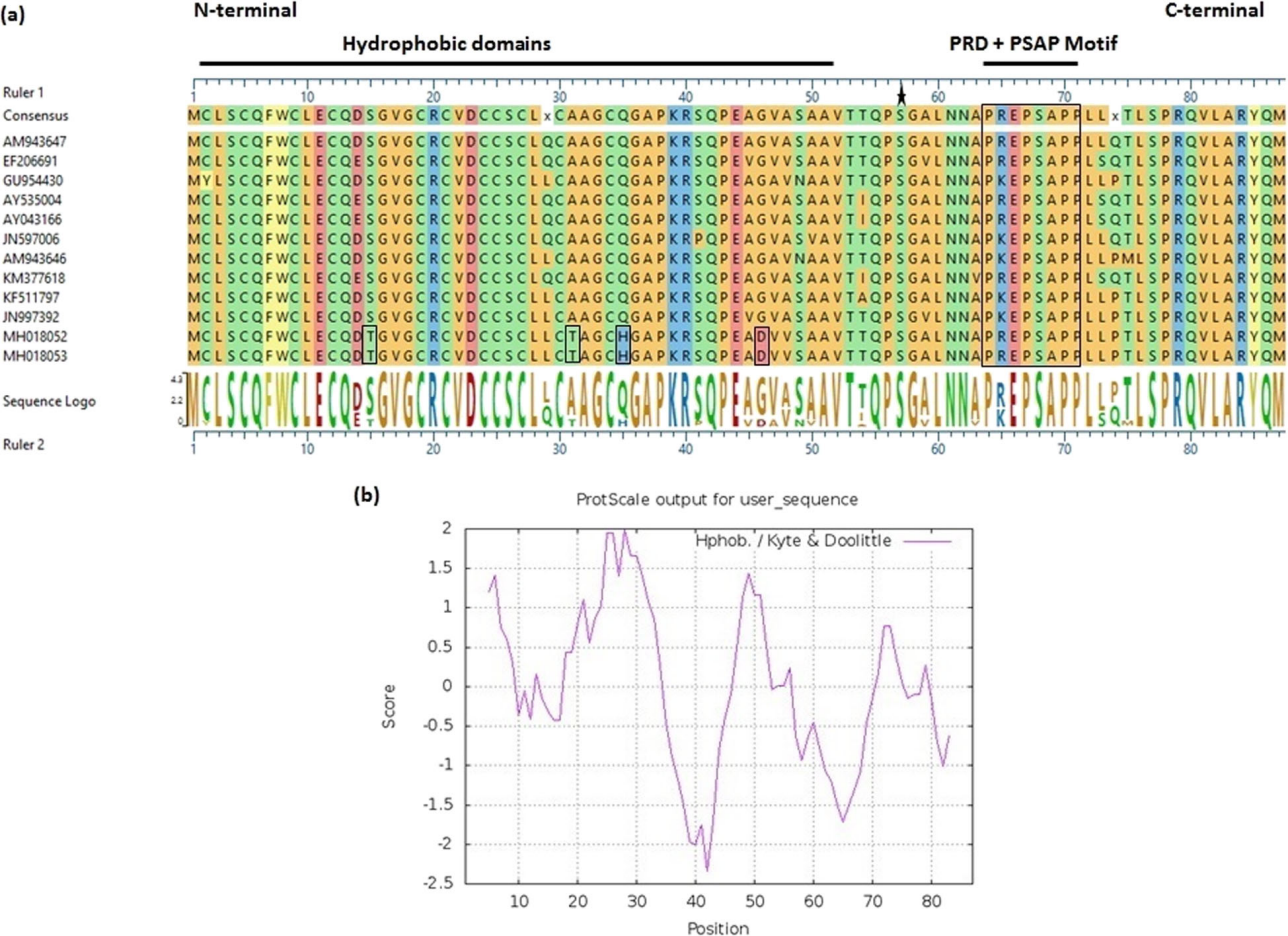
Molecular analysis for Pakistani aHEV ORF3 sequences. **a** Multiple sequence alignment showing conserved proline-rich domain (PREPSAPP) and unique amino acids for Pak aHEV sequences in the hydrophobic region (small boxes), **b** hydrophobicity plot for Pak aHEV sequences

Overall three hydrophobic domains (HD) were predicted in Pak aHEV ORF3 protein, two of which were located in N-terminal half, HD1 aa 3–10 and HD2 aa 16–38, and the third (HD3) almost in the middle of the protein, aa 43–50 (Fig. 6). The C-terminus contained a proline-rich domain (PRD), PREPSAPP (PXXPXXPP) (aa 64–74), with a single PSAP motif (aa 67–70). A conserved serine residue, adjacent to proline, located at position aa57 may be a potential phosphorylation site in the protein [23]. The N-terminal half was found to have 27.5% conserved cysteine residues across all aHEV sequences including the Pak aHEV sequences. In ORF3 the Pak aHEV sequences contained unique amino acid changes S15 T, A31T, Q35H, and G46D in the N-terminal half compared to other avian genotypes, (Fig. 6a).

## Discussion

In the present study, we isolated and identified two novel aHEV (Pak aHEV) strains from the bile fluid of two 71 week old layer chickens with clinical symptoms related to HSS [24]. The fecal samples from the chickens were negative for viral RNA, which may be due to collection of samples more than 8 weeks post-infection. The lack of detection of fecal virus shedding 8 weeks post-infection has been reported previously [7]. These cases seemed to be sporadic as there was no evidence of an outbreak in the area.

The Pak aHEV isolates analysis shared 98–100% nucleotide identity with each other across the sequenced regions. The NSI of partial ORF1, ORF2 and complete ORF3 with other aHEV sequences was 82–93%, which is comparable to the complete, or the nearly complete genome sequencing data of Moon et al. [25]. Similarly, NSI of 20–22% for Pak aHEV ORF3 sequences with *Orthohepevirus A* sequences are similar to results reported by Huang et al. [6]. The phylogenetic analysis, based on partial ORF1, ORF2 and complete ORF3, showed that the Pak aHEV sequences formed a monophyletic clade suggesting that Pak aHEV sequences belong to a previously unknown aHEV genotype [24]. Characterization of the ORF1/ORF3 intergenic region indicated two important conserved features in Pak aHEV sequences. First, the CRE sequence is thought to act as a promoter for the subgenomic bicistronic (ORF2, ORF3) RNA [26]. In contrast to little egret HEV [8], the CRE sequence of Pak aHEV strains did not include the ORF1 stop codon, which was found three nucleotides upstream in this study. Second, the SLS sequence is situated five nucleotides downstream of the CRE. There are five base changes in Pak aHEV compared with the other aHEV genotypes; however, the stem loop structure is maintained in the Pak aHEV SLS (Fig. 5a) [27, 28].

The importance of the OFR3 encoded protein as a multifunctional protein has been described in previous studies [29]. In addition, a recent study elucidated its function as a viroporin, a virus-encoded ion channel to facilitate release of infectious virions from the host cell [30]. In this context, two hydrophobic domains are found in the N-terminal half of the Pak aHEV ORF3 protein and a third is found in the middle of the protein. Hydrophobic domains have been identified in the N-terminus of other *Orthohepevirus* isolates [31]. The C-terminus of Pak aHEV strains contained a single proline-rich domain (PRD) as found among other *Orthohepevirus* isolates [7, 28]. Moreover, a single PSAP motif was found within the PRD, which has been implicated in viral egress through interaction with host tumor suppressor gene 101 (TSG101) protein and viroporin formation [7, 30]. The conserved serine residue in the PSAP motif found among *Orthohepevirus* isolates is also found in the Pak aHEV sequences, which may be a potential phosphorylation site for MAP kinase [32]. The N-terminal half of Pak aHEV ORF3 is hydrophobic, with a high proportion of cysteine residues (27.5%), which may reinforce ORF3 protein folding in the extracellular environment through disulfide bond formation when HEV virions egress the host cell, and act as metal binding motifs inside the host cell [22, 32, 33]. Pak aHEV ORF3 sequences contain unique amino acid changes S15 T, A31T, Q35H and G46D compared to other aHEV genotypes.

Limitations of our study include the fact that only two specimens could be sequenced. In addition there was not enough material to obtain a complete genome sequence from either specimen. For this reason we cannot prove these sequences belong to a novel genotype, even though they do not cluster in any known genotype, and the lack of complete genome sequences prevents us from proving these are unique strains.

To our knowledge, this study is the first report describing a novel avian HEV from Pakistan and possibly South Asia, providing further information for aHEV genetic diversity, genotype mapping, global distribution and epidemiology. Additionally, determining the prevalence of aHEV in broiler breeder and layer chickens in Pakistan may help further to characterize this strain and yield information about its impact on the health of the chicken population in Pakistan. This report is based on partial genome sequences; hence, further characterization based on complete genome sequences is needed to determine whether the Pakistani sequences may belong to a new aHEV genotype.

## Conclusions

On the basis of partial MeT, Hel, ORF2 and complete ORF3 sequences the present study reveals that Pak aHEV isolates may represent a novel Pakistani clade and high sequence homology to each other support the supposition they may belong to a monophyletic clade circulating in the region around Pakistan. Pak aHEV ORF3 sequences contain unique amino acid changes S15 T, A31T, Q35H and G46D compared to other aHEV genotypes. The data presented in this study provide further information for aHEV genetic diversity, genotyping, global distribution and epidemiology.

## Data Availability

Sequences have been submitted to GenBank with accession numbers MG692742, MG692743, MG692744, MH018052, MH018053, MH094852, MH094853, MH243320 and MH243321.

## Abbreviations

aHEV: Avian hepatitis E virus
BLS: Big Liver and Spleen
CRE: *Cis*-reactive element
Gt: Genotype
HD: Hydrophobic domain
Hel: Helicase
HSS: Hepatitis-splenomegaly syndrome
HVR: Hypervariable region
MeT: Methyltranferase
ORF: Open reading frame
Pak aHEV: Pakistani aHEV
PRD: Proline-rich domain
SLS: Stem-loop structure
